# Distinguishing between enduring and dynamic concussion symptoms: applying Generalisability Theory to the Rivermead Post Concussion Symptoms Questionnaire (RPQ)

**DOI:** 10.7717/peerj.5676

**Published:** 2018-09-28

**Authors:** Oleg N. Medvedev, Alice Theadom, Suzanne Barker-Collo, Valery Feigin, BIONIC Research Group

**Affiliations:** 1 School of Public Health and Psychosocial Studies, Auckland University of Technology, Auckland, New Zealand; 2 School of Medicine, University of Auckland, Auckland, New Zealand; 3 National Institute for Stroke and Applied Neuroscience, Auckland University of Technology, Auckland, New Zealand; 4 School of Psychology, University of Auckland, Auckland, New Zealand

**Keywords:** Rivermead Post Concussion Symptoms Questionnaire, Trait, Generalisability Theory, Measurement, Psychometrics, State

## Abstract

**Background:**

The Rivermead Post Concussion Symptoms Questionnaire (RPQ) is a widely-used, 16-item measure of concussion symptoms yet its ability to assess change in the symptom experience over time has come under criticism. We applied Generalisability theory to differentiate between dynamic and enduring aspects of post-concussion symptoms and to examine sources of measurement error in the RPQ.

**Materials and Methods:**

Generalisability theory was applied using the longitudinal design with persons as the object of measurement. Patients with a traumatic brain injury (*n* = 145; aged ≥16 years) were assessed at three time occasions (1, 6 and 12 months post-injury) using the RPQ.

**Results:**

The RPQ showed overall strong generalisability of scores (*G* = .98) across persons and occasions with a minor proportion of variance attributed to the dynamic aspect of symptoms reflected by interaction between person and occasion. Items measuring concentration, fatigue, restlessness and irritability reflected more dynamic patterns compared to more enduring patterns of sensitivity to noise, impatience, nausea and sleep disturbance.

**Conclusion:**

The RPQ demonstrated strong reliability in assessing enduring post-concussion symptoms but its ability to assess dynamic symptoms is limited. Clinicians should exercise caution in use of the RPQ to track dynamic symptom change over time. Further investigation is necessary to enhance the RPQ’s ability to assess dynamic symptoms and to address measurement error associated with individual items.

## Introduction

The Rivermead Post-concussive Symptom Questionnaire (RPQ; King et al., 1995) is a widely used clinical assessment tool designed to measure symptoms occurring after a traumatic brain injury (TBI). The RPQ is commonly used to measure severity of symptoms following mild or moderate traumatic brain injury by presenting 16 symptoms thought to be common consequences of such an injury. These symptoms, which include difficulties in cognition/thinking (e.g., memory, concentration), mood or affective complaints (e.g., depressed mood, irritability, anxiety), and somatic/physiological symptoms (e.g., dizziness, headache, fatigue, light sensitivity) are often referred to as “post concussive symptoms” (PCS) (McAllister, 2008). For each item the individual is asked to rate presence of the symptom over the previous 24 h compared with before the head injury. Symptoms are assessed on a five-point scale with the response alternatives: never had symptoms (Category 0), have had symptoms but they have resolved (Category 1), have mild problems with symptoms (Category 2), have moderate problems with symptoms (Category 3), and have severe problems with symptoms (Category 4). The total RPQ score is the sum of a subject’s score for each of the 16 items. Generally, PCS can be more enduring or dynamic, which depends on affected brain areas and damage severity (Carroll et al., 2004; Sveen et al., 2001). Enduring symptoms refer to symptoms that remain relatively stable over longer period of time (e.g., 6–12 months) while dynamic symptoms are unstable and may fluctuate substantially within days or months. Inability to distinguish clearly between enduring and dynamic symptoms may bias assessment of patients and lead to unreliable conclusions regarding their recovery and treatment effects.

The RPQ was originally designed to track individual symptoms and total symptom load in an individual, and over time with no inherent inclusion of subscales. A number of studies have examined performance on the RPQ to determine if the content of the scale could be divided into subscales for clinical use, with the findings being quite varied. This variability has been shown both across samples and across time within a given sample.

For example, in looking at variability across samples, a number of studies have examined the factor structure of the RPQ after TBI (Feigin et al., 2013; Aarons, Sklar & Sevdalis, 2017; Eyres et al., 2005; Franke et al., 2015). In a study of individuals 6 months post mild/moderate TBI, two- and three-factor models demonstrated equally good fit (*n* = 168; Potter et al., 2006). The three factor solution combines cognitive, affective, and somatic items into separate factors; while in the two-factor model split the items into cognitive symptoms and a single factor comprising both affective and somatic items. A different study which examined 2,602 individuals, assessed 3 months post mild TBI replicated the above finding, describing the same two- and three- factor structures, supporting the validity of these two models (Lannsjö et al., 2009).

Whilst the above two studies were consistent in their findings, other studies report different factor structures. In a study of individuals referred 3–6 months after TBI (*N* = 369; Eyres et al., 2005), the RPQ was split into two separate scales, the RPQ-3 (i.e., headaches, dizziness, nausea/vomiting) which is thought to reflect acute symptoms, and the RPQ-13 (i.e., fatigue, sleep disturbance, forgetful, depressed, concentration, irritable, slowed thinking, frustrated, restless, noise and light sensitivity, blurred vision, double vision) which is thought to reflect symptoms that can be either acute and/or enduring. Using Rasch analysis, each set of items formed a unidimensional construct, the two scales showed good test-retest reliability across a two week interval and adequate external construct validity.

Franke et al. (2015) examined the factor structure of the RPQ in a sample of military personnel following blast exposure (both with and without a known history of mild TBI). The findings suggest the RPQ has a four-factor structure, with factors interpreted as reflecting emotional, cognitive, visual, and vestibular functions. However, as there were no significant associations between a history of mild TBI and factor scores, the authors concluded that persistent PCS after blast exposure were related to four distinct forms of distress, but not to mild TBI *per se*. The above findings suggest that the RPQ has a varied structure depending upon the sample being examined.

Collectively, these findings indicate that there may be important clusters of more dynamic and more enduring symptoms within the post-concussion syndrome, which influences accuracy of assessment. Moreover, the measure may be affected by other sources of error not identified in the RPQ such as individual items, assessment occasions and their interaction with object of measurement (patients) (Medvedev et al., 2017). For instance, item-occasion interaction means that item wording/content may be interpreted differently at different occasions (e.g., ambulance/car vs home), creating measurement error. Rather than relying solely on factor analysis, the psychometric properties of the RPQ should be investigated using more advanced psychometric methods suitable to identify and evaluate various sources of measurement error and distinguish between dynamic and enduring aspects of symptoms. Effectiveness of a short-term intervention (i.e., 1–7 days) can be evaluated by measuring dynamic aspects of symptoms while effectiveness of a long-term treatment (i.e., 4–6 months) may be better assessed by measuring enduring symptom patterns (Medvedev et al., 2017). Clear distinction between dynamic and enduring symptoms may help clinicians and researchers to better measure and monitor symptom changes over time and to provide the most appropriate treatment at the relevant time point (Paterson et al., 2017).

The original paper presenting the RPQ as a measure investigated its inter-rater reliability (*r* = 0.91) as well as the test-retest reliability (*r* = .87; across 7 day interval) of the total score and the individual item scores (King et al., 1995). Using merely a correlation between total scale scores obtained at two different points of time is not an appropriate approach to estimate test-retest reliability and the intraclass correlation coefficient (ICC) should be used for this purpose. If a patient scores 4 on concentration, 1 on restlessness and 2 on noise sensitivity in the first month and then 1 on concentration, 4 on restlessness and 2 on noise sensitivity in the next month the total score remains the same (e.g., 7) resulting in full agreement between two assessments with 1-month interval, which does not reflect clinically important PCS change. Note that in this example concentration and restlessness appear as dynamic symptoms while noise sensitivity as an enduring symptom. Therefore, the use of the total score rather than the item scores does not permit accurate estimation of reliability over time and clear distinction between items measuring dynamic and stable symptoms. Moreover, the reliability estimated based on the total score does not account for measurement error due to item, occasion and their interactions with object of measurement (person) (Medvedev et al., 2017; Bloch & Norman, 2012). Generalisability Theory (G Theory) was proposed as suitable method for demonstrating distinction between stable (trait) and dynamic (state) components in a measure and thoroughly evaluating all major sources of error affecting measurement (Medvedev et al., 2017; Shavelson, Webb & Rowley, 1989). A trait is usually defined as a relatively enduring or stable characteristic of a person while a state refers to characteristic displayed in a given situation or moment. A state is a dynamic characteristic and results from interaction between person (trait) and occasion, which is the organism’s unique adaptation to the momentary environment (Spielberger, Gorsuch & Lushene, 1970). Reliable distinction between enduring and temporary symptom patterns is an important clinical issue especially if evaluating recovery from TBI because temporary changes (e.g., mood) could affect accuracy of diagnosis leading to inappropriate treatment. Clinically applied measures should distinguish clearly between state and trait aspects of a person’s presentation and account for the relevant sources of measurement error, which needs to be established using an appropriate psychometric technique such as G Theory (Paterson et al., 2017; Bloch & Norman, 2012).

G Theory is a statistical theory developed by Cronbach that provides distinct advantage over Classical Test Theory methods for evaluating reliability of psychometric instruments and that enables us to disentangle specific sources of measurement error (Cronbach, Rajaratnam & Gleser, 1963) and distinguish between stable and dynamic components in a measure (Medvedev et al., 2017; Paterson et al., 2017). We favoured G Theory approach for the current study because compared to the other available methods (e.g., Hamaker, Nesselroade & Molenaar, 2007; Geiser et al., 2015; Kenny & Zautra, 2001) that are used to distinguish state from trait it is also a well-established method to evaluate reliability of psychometric instruments and identify specific sources of measurement error. The aim of the current study was to apply G Theory to differentiate between dynamic and enduring aspects of post-concussion symptoms and to examine sources of measurement error in the RPQ. G theory was applied using the longitudinal design with persons as the object of measurement. Patients with a traumatic brain injury (*n* = 145); aged ≥16 years) were assessed at three time occasions (1, 6 and 12 months post-injury) using the RPQ. G Theory involves two parts: Generalisability study (G-study) examined the generalisability of the RPQ scores and sources of measurement error in the current measurement design, followed by a Decision study (D-study) to explore psychometric properties of the measure by manipulating measurement design (i.e., the factor structure) to optimise reliability (Shavelson, Webb & Rowley, 1989; Cardinet, Johnson & Pini, 2010).

## Materials & Methods

### Study population

The sample for this study was extracted from a longitudinal TBI cohort study for which the methodology and findings have been published separately (Feigin et al., 2013; Theadom et al., 2012). Within the main study, all cases of TBI that occurred in the Hamilton and Waikato Districts of New Zealand (NZ) during a 1 year period (1 March 2010 through 28 February 2011) were identified using both community (e.g., sports clubs, prisons, and schools) and medical services (e.g., Hospitals/Emergency Clinics, General Practitioners and allied health professionals).

TBI was defined as an injury to the brain resulting from an external force to the head in accordance with the World Health Organisation criteria (Carroll et al., 2004). Medical records and self-reported information for all potentially eligible TBI cases was reviewed by a diagnostic review group including experienced neurologists and neuropsychologists to ensure that each case met the inclusion criteria/definition of TBI.

All confirmed TBI cases (*n* = 1,369) were invited to complete an assessment of the impact of their TBI at 1, 6 and 12 months following injury to monitor their recovery. Assessments were completed in person at the participant’s place of residence or at another mutually convenient location (e.g., private room at a GP practice, library or university). While the main study included people of all TBI severities, the purpose for the current analysis was to explore utility of the RPQ for assessing symptoms following mild TBI. Therefore, the full cohort (*n* = 870; Table 1) included only cases classified as being of mild severity (i.e., Glasgow Coma Score of 13–15 and/or Post-traumatic Amnesia < 24 h) and only adult cases (those aged ≥16 years) because 16 is the lower limit for the RPQ test. The incidence study identified 145 patients (17% of the full cohort) with mild TBI (cases aged ≥16 years), who provided responses without missing data for all three post-injury assessments, and only these data were included in this analysis (Table 1).

**Table 1:** Demographic characteristics of the current sample and those not completing the RPQ on all three occasions.

	Current sample *N* = 146	Full Cohort *N* = 870	Test of difference
Mean Age (SD)	39.7 (18.0)	37.4 (19.3)	*p* = 0.17^a^
Sex *n* (%)			
Male	86 (58.9)	532 (61.1)	*p* = 0.61^b^
Female	60 (41.1)	338 (38.9)	
Ethnicity *n* (%)			
European	101 (69.2)	544 (62.5)	*p* = 0.09^b^
Mãori	40 (27.4)	254 (29.2)	
Other	5 (3.4)	72 (8.2)	
Additional Injuries *n* (%)			
Yes	108 (74.0)	612 (70.3)	*p* = 0.37^b^
No	38 (26.0)	258 (29.7)	
Intentional Injury *n* (%)			
Yes	32 (21.9)	193 (22.2)	*p* = 0.94^b^
No	114 (78.1)	677 (77.8)	

**Notes.**

a *t-* test.

b *χ*^2^ test.

### Procedure

The study was approved by the Auckland University of Technology Ethics Committee (09/265) and the Northern Y Health and Disability Ethics Committee of NZ (NTY/09/09/095). Written informed consent was obtained from all study participants. Data was collected within 1 month, 6- and 12-months post injury and the procedure of data collection is described in more details elsewhere (Barker-Collo et al., 2016).

### Statistical Analyses

Prior to the main analyses demographic characteristics of the full cohort and extracted sample were compared using t-tests (e.g., age) and Chi-square tests. We have screened data for normality of distribution, calculated Cronbach’s alpha coefficients individually for each assessment occasion, and estimated test-retest reliability using both ICC for all three occasions and Pearson’s r correlation between the baseline assessment and 6 and 12 months post-injury. The RPQ summed scores were computed by adding individual items scores at each occasion and *t*-test comparisons were conducted between the baseline and 6 and 12 months assessments.

G Theory was applied following detailed recommendations described elsewhere (Cardinet, Johnson & Pini, 2010) and using EduG 6.1–e software (Swiss Society for Research in Education Working Group, 2006). G theory is applied in four sequential steps including defining the measurement design (1); estimating variance components by applying traditional ANOVA (2); computing the overall reliability (G-coefficient) of the RPQ and estimating sources of measurement error using the ANOVA results in a G-study (3); and conducting a D-study to calculate variance estimates and G-coefficients for different measurement designs to optimise reliability of the instrument (4).

Step 1: We used repeated measures ANOVA with 2 levels (facets) random effects measurement design defined as person (P), by item (I), by occasion (O) expressed as P × I × O, where the P and O are random and I is fixed to the number of items. The object of measurement were persons (differentiation facet), which is not considered as a source of error, and items and occasions were defined as instrumentation facets (Cardinet, Johnson & Pini, 2010). This measurement design was specified in EduG as P/IO with the following (145 ×16 ×3). Interaction between person and occasion (P ×  O) reflects a state or dynamic component in a measurement and can be used to estimate scale sensitivity to state changes represented as the State Component Index (SCI) (Medvedev et al., 2017). Definitions of components for both generalisability (G-) and decision (D-) studies using two-facet design are included in Table 2.

**Table 2:** Components definitions for Generalizability study with two-facets (P ×  I ×  O).

Persons (P)	Universe of person scores *p* (averaged deviation of individual scores from grand mean over items and occasions)
Items (I)	Item effect *i* (averaged deviation of item scores from grand mean over persons and occasions)
Occasions (O)	Occasion effect *o* (averaged deviation of occasion scores from grand mean over persons and items)
P × I	Effect of interaction between person *p* and item *i* averaged over occasions
P × O	Effect of interaction between person *p* and occasion *o* averaged over items
P × I × O, *e*	Effect of interaction between person *p*, item *i* and occasion *o,* containing random error

Step 2: Traditional ANOVA was used to compute variance components due to person (P), item (I), occasion (O) and interactions between these facets. EduG software accurately estimates variance components by using Whimbey’s correction coefficient (Cardinet, Johnson & Pini, 2010) expressed as (N(f) −1)/N(f), where N(f) is the population size of the f facet in the G-study design that has no effect on facets derived from infinite populations (e.g., persons) but considers finite facets such as items.

Step 3: The G-study separates object of measurement (person) from other facets to compute variance components for each facet together with their interactions and generalisability (G-) coefficients for the object of measurement (person) using equations developed by Brennan (2001). There are relative and absolute G-coefficients computed by EduG, relative G-coefficient (*ρ*^2^ or ϖ^2^) only accounts for variance directly associated with the object of measurement (Brennan, 2001; Gardinet, Johnson & Pini, 2009). The absolute G-coefficient or Phi (Φ) considers other sources of variance (e.g., item × occasion interaction) that may affect absolute measurement indirectly (Gardinet, Johnson & Pini, 2009). In this paper, we refer to the absolute G-coefficient as G-coefficient because it is a more accurate and conservative measure of reliability (Bloch & Norman, 2012). Generally, a higher G-coefficient (e.g., >.80) is characteristic of a trait measure (Arterberry et al., 2014). SCI was computed using formula developed by Medvedev et al. (2017). The full 16-item RPQ was subjected to the G-study analysis.

Step 4: D-Study examined variance components associated with the object of measurement and individual facets by manipulating facet design to optimise reliability of measurement. It includes individual item and subscale analyses to evaluate reliability of proposed factor structures. A number of potential RPQ models were tested. In the first model, RPQ somatic, cognitive and affective symptom clusters were examined along with a combined cognitive + affective cluster (Potter et al., 2006). In the second model the first three symptoms of RPQ (headaches, nausea/vomiting, dizziness) are referred to as RPQ-3 or RPQh (RPQ head), and are thought to represent the early (within 2 weeks of injury) symptoms associated with post-concussion syndrome; whilst the remaining 13 items (RPQ-13) are thought to reflect symptoms that are more likely to persist (Sveen et al., 2001).

## Results

Table 1 compares demographic characteristics of the full cohort and extracted sample indicating no significant differences in demographic characteristics between the samples. Descriptive statistics for individual items, occasions and the RPQ total score together with Cronbach’s alpha coefficients and test-retest reliability scores are presented in Table 3. RPQ demonstrated strong internal consistency across all three occasions (*α* = .94) but test-retest *r*-scores compared to the baseline were slightly below .60 (CI ±.10) and ICC for all three occasions was slightly higher at .63 (CI ± .08). Overall item mean score did not differ significantly across occasions, but a significant decrease in the summed RPQ score was observed after 6 and 12 months compared to the baseline. However, the difference between total mean scores at 6 and 12 months was not statistically significant. Table S1 includes measures of central tendency for distribution of the RPQ items across three occasions and shows that most of the items scores satisfy conservative criteria for normal distribution with skewness and kurtosis values within range of ±1, except of items 3, 8 and 15. Median and quartile range measures indicating the overall positive skewness of the item data with the 1st quartile score of 0 and median range from 0 to 1 for all the items.

**Table 3:** Descriptive statistics including mean, standard deviation (SD), Cronbach’s alpha (*α*) at each occasion and test–retest reliability for the RPQ (*n* = 145 × 3 occasions).

Item	Mean	SD	*α*	Test–retest (ICC, r)
1. Headaches	1.2	1.2		
2. Feelings of dizziness	1.0	1.1		
3. Nausea and /or vomiting	0.4	0.8		
4. Noise sensitivity	0.8	1.1		
5. Sleep disturbance	1.2	1.2		
6. Fatigue, tiring more easily	1.4	1.3		
7. Irritable, easily angered	1.0	1.1		
8. Depressed or tearful	0.7	1.0		
9. Frustrated or impatient	1.1	1.1		
10. Forgetful, poor memory	1.4	1.2		
11. Poor concentration	1.2	1.2		
12. Taking longer to think	1.3	1.2		
13. Blurred vision	0.8	1.1		
14. Light sensitivity	0.8	1.2		
15. Double vision	0.4	0.8		
16. Restlessness	1.0	1.1		
Occasion				
1 (Baseline/within 1 month)	1.0	1.2		
2 (6 months post-injury )	1.0	1.2		
3 (12 months post-injury)	1.0	1.2		
RPQ 16 Items summed score				(ICC) .63(CI ± .08)
1 (Baseline/within 1 month)	19.5	13.6	.94	–
2 (6 months post-injury)	14.4^*^	12.5	.95	(*r*) .57(CI ± .10)
3 (12 months post-injury)	12.9^*^	12.2	.94	(*r*) .56(CI ± .10)

**Notes.**

Note: Grand mean, 1.0; SD, 1.2; ICC, Intraclass Correlation Coefficient across three occasions; *r*, Pearson’s correlation between baseline (1) time 2 and 3; CI, 95% Confidence Interval.

* Significant mean difference compared to the baseline using paired *t*-test *p* < .001.

### G-study

Traditional ANOVA estimates for person (P), item (I), occasion (O) and their interactions are presented in Table 4 (column 7) and were used to compute variance components in the G-study that accurately reflect a unique contribution of each potential source of error variance. Unlike traditional ANOVA, in a G-study all error estimates are computed individually after excluding person or discrimination variance, which is the object of measurement and not a source of error (Table 5).

**Table 4:** ANOVA table for the RPQ using Person (P) ×  Item (I) ×  Occasion (O) design with interactions (*n* = 145).

				Variance Components				
Source	SS	df	MS	Random	Mixed	Corrected^a^	%	SE^b^
P	2,984.61	144	20.73	0.40	0.42	0.42	30.0	0.05
I	249.50	15	16.63	0.00	0.00	0.00	0.2	0.02
O	0.10	2	0.05	−0.01	0.00	0.00	0.0	0.00
P × I	3,038.50	2,160	1.41	0.28	0.28	0.28	19.8	0.01
P × O	145.86	288	0.51	0.00	0.03	0.03	2.3	0.00
I × O	442.54	30	14.75	0.10	0.10	0.10	7.0	0.03
P × I × O	2,470.84	4,320	0.57	0.57	0.57	0.57	40.8	0.01
Total	2,108.32	6,959					100%	

**Notes.**

SS sum of squares df degrees of freedom MS mean squares variance components (in %) SE standard errors

a Corrected components are calculated by applying Whimbey’s correction to the ANOVA estimates.

b SE in the right column is related to the mixed effects presented in column 6.

Table 5 shows that after accounting for all sources of error RPQ scores have good generalisability across universe of patients with mild TBI and post-injury occasions with an absolute G-coefficient of .98 that accounts for all sources of error identifiable in the data (Brennan, 2001; Arterberry et al., 2014). Interaction between person and occasion reflects dynamic component or individual state (Medvedev et al., 2017) and accounts for all remaining variance in RPQ after accounting for enduring person patterns (Table 5). However, this dynamic variance component is relatively small compared to variance attributed to enduring aspect of symptoms (SCI = .02). This indicates that the scale is not sensitive to dynamic aspects of symptoms and predominantly measures enduring symptom patterns.

### D-study

Individual facet analyses were conducted for every item and subscales consistently identified by factor analyses of earlier studies with G-coefficients, the relevant variance components and SCIs presented in Table 6. Absolute G-coefficients that reflect enduring aspects in a measure are presented in descending order for individual items. Items measuring concentration, fatigue, restlessness, irritability, headache and taking longer to think were more sensitive to dynamic symptom patterns (G range .60–.69), which is reflected by higher SCI (.28–.33). Items measuring more enduring symptom characteristics included sensitivity to noise, impatience, nausea and sleep disturbance (G range .82–.92; SCI range .07–.16). All individual subscales had a G-coefficient above .81 indicating good generalisability of scores for measurement of stable trait-like symptoms and lack of sensitivity to dynamic symptoms (SCI ≤ .03). Cognitive, somatic and RPQ-3 (headache, nausea, dizziness) subscales that were most affected by error involved interaction of item and person suggesting that they contain items contributing to undesired measurement error. The RPQ-13 was the most reliable subscale in measuring enduring symptom patterns (G = .96) that was not affected by error associated with individual items.

**Table 5:** Estimated variance components with standard errors (SE) and G-coefficients for the RPQ G-study P/IO design (*n* = 145).

Source of variance	Differentiation variance	Relative error variance	% relative	Absolute error variance	% absolute
P	0.42	.....		.....	
I	.....	.....		(0.00)	0.0
O	.....	.....		(0.00)	0.0
P × I	.....	(0.00)	0.0	(0.00)	0.0
P × O	.....	0.01	100.0	0.01	100.0
I × O	.....	.....		(0.00)	0.0
P × I × O	.....	(0.00)	0.0	(0.00)	0.0
Sum of variances	0.42	0.01	100%	0.01	100%
Standard deviation	0.65	Relative SE:	0.10	Absolute SE:	0.10
Coeficient G relative	0.98
Coeficient G absolute	0.98

**Notes.**

Grand mean = 0.98; SE of the grand mean: 0.05.

**Table 6:** Variance components of person (P), occasion (O) and P × O interaction together with absolute and relative G-coefficients and state component index (SCI) for each individual item of the RPQ (*n* = 145 × 3).

**Items**	**Variance components**				**G**	**G**	
	P	O	P × O	P × I	**Relative**	**Absolute**	**SCI**
4. Noise sensitivity	1.24	0.00	0.10	–	0.93	0.92	0.07
9. Frustrated or impatient	0.85	0.00	0.14	–	0.86	0.86	0.14
3. Nausea and /or vomiting	0.78	0.02	0.14	–	0.85	0.83	0.15
5. Sleep disturbance	0.72	0.02	0.14	–	0.84	0.82	0.16
8. Depressed or tearful	0.80	0.04	0.17	–	0.82	0.79	0.18
10. Forgetful, poor memory	0.70	0.03	0.18	–	0.79	0.77	0.20
13. Blurred vision	0.74	0.02	0.20	–	0.78	0.77	0.21
2. Feelings of dizziness	0.86	0.03	0.26	–	0.77	0.75	0.23
15. Double vision	0.71	0.03	0.20	–	0.78	0.75	0.22
14. Light sensitivity	0.66	0.05	0.18	–	0.79	0.74	0.21
12. Taking longer to think	0.54	0.03	0.21	–	0.72	0.69	0.28
1. Headaches	0.58	0.06	0.21	–	0.74	0.69	0.27
7. Irritable, easily angered	0.46	0.05	0.19	–	0.71	0.66	0.29
16. Restlessness	0.41	0.03	0.22	–	0.65	0.62	0.35
6. Fatigue, tiring more easily	0.42	0.01	0.26	–	0.62	0.61	0.38
11. Poor concentration	0.45	0.08	0.22	–	0.68	0.60	0.33
**Subscales/factors**							
Somatic	0.45	0.00	0.01	0.02	0.93	0.92	0.02
Cognitive	0.48	0.00	0.00	0.03	0.84	0.81	0.00
Affective	0.49	0.00	0.01	0.01	0.90	0.89	0.02
Cognitive +Affective	0.48	0.00	0.01	0.01	0.93	0.92	0.02
RPQ-13	0.44	0.00	0.01	0.00	0.96	0.96	0.02
RPQ-3	0.66	0.00	0.02	0.03	0.86	0.85	0.03

**Notes.**

a P ×  I variance components are not available for single item/level analysis. Absolute G-coefficient is presented in descending order for individual items.

## Discussion

The study results show good generalisability of the RPQ (G = .98) indicating that the instrument can reliably be used across wide TBI populations and occasions to measure trait-like or enduring concussion symptoms only (Arterberry et al., 2014). The RPQ was found unsuitable for measuring dynamic state-like symptoms (Medvedev et al., 2017; Paterson et al., 2017). This limits the instrument’s applicability for monitoring of patients condition over time and other more sensitive tools need to be developed in order to assess dynamic state-like symptoms. Low sensitivity to dynamic symptoms demonstrated by the RPQ in this study supported by mean comparisons showing no significant differences within a half a year period between 6 and 12 months assessments. Enduring concussion symptoms change normally occurrs within the first 6 months (Feigin et al., 2013; Carroll et al., 2004; Sveen et al., 2001) and was reflected by the RPQ showing significant mean difference between baseline and both 6 and 12 month assessments.

We note that cognitive, somatic and RPQ-3 (headache, nausea, dizziness) subscales previously identified in the literature were most affected by measurement error associated with items, and even after accounting for error these scales had acceptable generalisability (G > .81) for measuring enduring symptoms. Consistent with earlier Rasch analysis (Eyres et al., 2005) the most reliable subscale identified in D-study was RPQ-13 (G = .96) that displayed no significant error associated with individual items. This subscale does not include RPQ-3 items measuring headache, nausea and dizziness and further research is needed to improve psychometric properties of these items. One item measures both nausea and/or vomiting at the same time, which may or may not co-occur and that may bias the measurement. Items measuring sensitivity to noise, impatience, nausea and sleep disturbance displayed higher G-coefficients and lower SCIs suggesting that these concussion symptoms have more enduring characteristics. Items measuring concentration, fatigue, restlessness, irritability, headache and taking longer to think had G-coefficients below .70 and SCIs above .28 indicating the more dynamic nature of these symptoms.

Relatively weak test-retest reliability scores of .57 and .56 at 6 and 12 months respectively, compared to the baseline are consistent with that reported earlier (Paterson et al., 2017) and may reflect limitations of correlational method because it does not account for change at individual item level and only compares two occasions at a time. However, we also calculated ICC that accounts for all three occasions simultaneously and overcomes the second limitation of Pearson’s correlation mentioned here resulting in slightly higher reliability estimate (.63). Relatively low ICC score may reflect natural reduction of PCS over time (Feigin et al., 2013; Carroll et al., 2004; Sveen et al., 2001).

It should also be noted here that many of the symptoms contained in the RPQ, and particularly those such as nausea, headache, dizziness, and fatigue, are common within a general population (e.g., due to minor illness, associated with alcohol use, etc.) and therefore it is not unlikely that they would fluctuate (Sawchyn, Brulota & Strauss, 2000). The literature suggests that endorsement of PCS symptoms occurs with considerable frequency in the normal population, and particularly in individuals with medical or psychological problems, and individuals involved in litigation (Fox et al., 1995; Lees-Haley & Brown, 1993). Reliable and valid measure of PCS should account for this, which is only possible if a measure can distinguish between more enduring and temporary fluctuating symptoms. Such distinction is well established between state and trait anxiety and a robust measurement tool the State-Trait Anxiety Inventory is widely used for such assessments (Spielberger, Gorsuch & Lushene, 1970). Further research is necessary to establish distinction between state and trait aspects of PCS and reliable measurement of each aspect.

The accurate distinction between dynamic and stable symptoms is an essential step for establishing reliability and validity of health outcome measures. This study is novel because it used the most appropriate psychometric method–G Theory to evaluate reliability of the RPQ and to derive an empirical evidence to distinguish between enduring and more dynamic PCS (Hamaker, Nesselroade & Molenaar, 2007; Geiser et al., 2015; Medvedev et al., 2017). G Theory provides an advanced method for assessing various factors such as assessment items, occasions and their interactions that may potentially affect reliability, which contributes to the improvement of assessment methodology and precision of measurement. The findings of this study can be used by future studies to develop a measure that separately and reliably assesses dynamic and enduring PCS. Development of such a measure based on the current evaluation of PCS would practically improve clinical care by allowing monitoring of patients condition over time using dynamic symptoms measure and evaluating the overall severity of TBI impairment using enduring symptoms measure.

Strengths of this study were applying a robust method such as G Theory to distinguish between stable and dynamic symptoms and prospective population-based design to capture the initial sample, which ensured most complete case ascertainment at a community level. This resulted in a large study sample which included people often excluded from outcome studies of TBI (e.g., those not seeking medical treatment). The study also used standard criteria for reporting the results to allow international comparisons. However, generalisability of the study findings may be limited because only 17% of the full cohort who provided data at all three time points were included in the analysis. Another limitation of the study was that we also did not have enough number of TBIs for separate ethnic groups (e.g., European, Mãori, Pacific-Islanders, Chines, South Asians, etc.), who might perform differently on measures such as the RPQ due to cultural factors.

## Conclusion

The RPQ demonstrated good reliability in assessing enduring post-concussion symptoms, but its ability to assess dynamic symptoms is limited. Similarly, examination of existing methods for deriving subscales for the RPQ suggests that cognitive, somatic and RPQ-3 subscales are subject to considerable error and should not be used clinically. Cognitive and somatic symptoms may be related both neurologically and psychologically and assessing them separately may impact on reliability. The RPQ-3 may not work well psychometrically due to low number of items that are not representative of a separate PCS trait. Clinicians should use caution in applying of the RPQ to track symptom change over time particularly for dynamic symptoms such as concentration, fatigue, restlessness, irritability, headache and taking longer to think. Further investigation of the RPQ is necessary to address measurement error associated with individual items.

##  Supplemental Information

10.7717/peerj.5676/supp-1 Table S1 Measures of central tendency for distribution of the RPQ items across 3 occasions (*n* = 145)

10.7717/peerj.5676/supp-2 Supplemental Information 2 EduG G Analyses file 145 persons × 16 items × 3 Occasions (P/IO) Measurement design This EduG file includes imported data (145 × 16 × 3) and specified measurement design (P/IO) and can be opened from EduG interface to replicate findings of G and D studies of the PRQ.

10.7717/peerj.5676/supp-3 Supplemental Information 3 Participants (*n* = 145) scores on the 16 item RPQ (columns 1–16 corresponding to RPQ items 1–16) over 3 time points stacked The first row numbered 1–16 indicate the corresponding RPQ items (1–16). Data entries start from the second row.

## Additional Information and Declarations

### Data Availability

The following information was supplied regarding data availability: The raw data are provided in a Supplemental File.
