# Supplementary material for: Distinguishing between enduring and dynamic concussion symptoms: applying Generalisability Theory to the Rivermead Post Concussion Symptoms Questionnaire (RPQ)
Source: PeerJ. 2018 Sep 28;6:e5676. doi: 10.7717/peerj.5676 (PMC6166625; doi:10.7717/peerj.5676)
Supplement: Table S1 [file peerj-06-5676-s001.docx]

Supplementary Table 1S. Measures of central tendency for distribution of the RPQ items across 3 occasions (n=145).

| Measures | RPQ Items | 1 | 2 | 3 | 4 | 5 | 6 | 7 | 8 | 9 | 10 | 11 | 12 | 13 | 14 | 15 | 16 |
| --- | --- | --- | --- | --- | --- | --- | --- | --- | --- | --- | --- | --- | --- | --- | --- | --- | --- |
| Skewness |  | 0.77 | 0.83 | 2.13 | 1.10 | 0.87 | 0.48 | 0.98 | 1.45 | 0.85 | 0.54 | 0.77 | 0.55 | 1.30 | 1.33 | 2.46 | 1.03 |
| SE of Skewness | | 0.12 | 0.12 | 0.12 | 0.12 | 0.12 | 0.12 | 0.12 | 0.12 | 0.12 | 0.12 | 0.12 | 0.12 | 0.12 | 0.12 | 0.12 | 0.12 |
| Kurtosis |  | -0.40 | -0.49 | 4.03 | 0.00 | -0.19 | -0.90 | 0.16 | 1.50 | -0.07 | -0.61 | -0.35 | -0.68 | 0.78 | 0.75 | 5.62 | 0.30 |
| SE of Kurtosis | | 0.23 | 0.23 | 0.23 | 0.23 | 0.23 | 0.23 | 0.23 | 0.23 | 0.23 | 0.23 | 0.23 | 0.23 | 0.23 | 0.23 | 0.23 | 0.23 |
| Quartile 1 | 25 | 0 | 0 | 0 | 0 | 0 | 0 | 0 | 0 | 0 | 0 | 0 | 0 | 0 | 0 | 0 | 0 |
| Median | 50 | 1 | 0 | 0 | 0 | 1 | 1 | 1 | 0 | 1 | 1 | 1 | 1 | 0 | 0 | 0 | 1 |
| Quartile 3 | 75 | 2 | 2 | 0 | 2 | 2 | 2 | 2 | 1 | 2 | 2 | 2 | 2 | 1 | 1 | 0 | 2 |
